# Investigating the Mechanism of Horseradish Peroxidase as a RAFT-Initiase

**DOI:** 10.3390/polym10070741

**Published:** 2018-07-05

**Authors:** Alex P. Danielson, Dylan Bailey Van-Kuren, Joshua P. Bornstein, Caleb T. Kozuszek, Jason A. Berberich, Richard C. Page, Dominik Konkolewicz

**Affiliations:** 1 Department of Chemistry and Biochemistry Miami University 651 E High St, Oxford, OH 45056, USA; danielap@miamioh.edu (A.P.D.); baileydk@miamioh.edu (D.B.V.-K.); bornstj@miamioh.edu (J.P.B.); kozuszct@miamioh.edu (C.T.K.); pagerc@miamioh.edu (R.C.P.); 2 Department of Chemical, Paper and Biomedical Engineering Miami University 650 E High St, Oxford, OH 45056, USA; berberj@miamioh.edu

**Keywords:** RAFT polymerization, enzymatic polymerization, reaction kinetics, horseradish peroxidase, polymerization mechanism

## Abstract

A detailed mechanistic and kinetic study of enzymatically initiated RAFT polymerization is performed by combining enzymatic assays and polymerization kinetics analysis. Horseradish peroxidase (HRP) initiated RAFT polymerization of dimethylacrylamide (DMAm) was studied. This polymerization was controlled by 2-(propionic acid)ylethyl trithiocarbonate (PAETC) in the presence of H_2_O_2_ as a substrate and acetylacetone (ACAC) as a mediator. In general, well controlled polymers with narrow molecular weight distributions and good agreement between theoretical and measured molecular weights are consistently obtained by this method. Kinetic and enzymatic assay analyses show that HRP loading accelerates the reaction, with a critical concentration of ACAC needed to effectively generate polymerization initiating radicals. The PAETC RAFT agent is required to control the reaction, although the RAFT agent also has an inhibitory effect on enzymatic performance and polymerization. Interestingly, although H_2_O_2_ is the substrate for HRP there is an optimal concentration near 1 mM, under the conditions studies, with higher or lower concentrations leading to lower polymerization rates and poorer enzymatic activity. This is explained through a competition between the H_2_O_2_ acting as a substrate, but also an inhibitor of HRP at high concentrations.

## 1. Introduction

Enzymes are fundamental to biological processes due to their ability to efficiently catalyze reactions [1]. These same catalytic properties have applications in chemistry and biochemistry and can be used to synthesize complex molecules and materials [2,3]. Of particular interest is the concept of using enzymes to catalyze polymerization reactions and allow for polymer synthesis at faster reaction rates and under mild conditions [4,5,6,7]. This takes advantage of the efficiency of enzymes albeit for a new, non-native function, such as synthetic chemistry. The discovery of new enzyme applications for polymerization and the optimization of these processes through mechanistic and kinetic studies offer potential environmental and economic benefits, which makes them significant areas of interest [8].

Free radical polymerization is a commonly used polymerization technique that allows for the synthesis of a broad range of materials [9,10]. This type of polymerization involves a radical-producing initiation step, which can come from various sources including thermal initiators, photochemical processes, and can be catalyzed by a variety of enzymes such as horseradish peroxidase [11,12,13,14,15,16,17,18,19,20,21,22,23]. However, free radical polymerization has certain limitations, such as poor control over polymer microstructure and broad molecular weight distributions [24]. Reversible-deactivation radical polymerization (RDRP) represents an alternative type of polymerization that allow for the synthesis of well-defined polymers and is compatible with a wide range of functional groups [25,26]. RDRP methods include nitroxide-mediated radical polymerization (NMP), atom-transfer radical polymerization (ATRP), and reversible addition-fragmentation polymerization (RAFT) [27,28,29,30]. Metalloenzymes have been demonstrated as efficient initiators for RDRP processes, especially those that follow the ATRP-like mechanism [17,31,32,33,34,35]. This is in addition to the deoxygenation processes facilitated by enzymes such as glucose oxidase to promote polymerization under simple conditions [36,37,38,39].

**Scheme 1:**
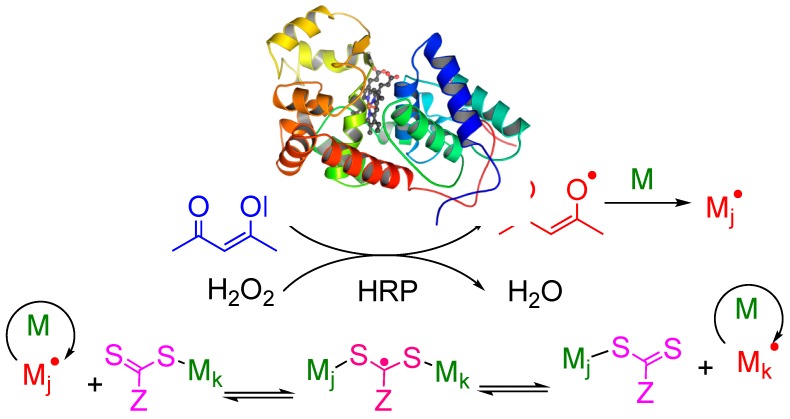
Top: enzymatic carbon centered radical generation and initiation of polymerization, and bottom: RAFT equilibrium and degenerative transfer.

RAFT is a prominent RDRP variant which offers distinct advantages such as compatibility with a wide range of functional groups and the ability to be run under simple and near ambient conditions [40]. Horseradish peroxidase (HRP) has been shown as an effective initiator for RAFT polymerization [39,41,42,43,44], making HRP a RAFT-initiase or an enzyme capable of initiating RAFT reactions. Horseradish peroxidase catalyzes the generation of free radicals from hydrogen peroxide [45]. Acetylacetone (ACAC) is used as a radical mediator, which transfers the radical to the monomer, which can then enter the RAFT equilibrium. Unlike ATRP processes that use HRP and other metalloproteins as catalysts [31,32,33,34,35], RAFT has a potential advantage in enzymatic RDRP. This is because a well-controlled RAFT process requires the enzyme to catalyze radical generation, but not radical deactivation back to the dormant state, since control is attained through the RAFT degenerative transfer equilibrium [46,47]. In contrast, enzymatic ATRP processes require the enzyme to be responsible for both chain-end activation and radical deactivation. In the RAFT processes initiated using an enzyme, a chain transfer agent (CTA) such as (2-propionic acid)yl ethyl trithiocarbonate is used to facilitate the uniform propagation of polymers as the monomers are added to the living chains in a controlled fashion. HRP initiated RAFT has been demonstrated as a rapid and versatile polymerization technique capable of synthesizing well-defined homopolymers and complex architecture such as block copolymers, protein-polymer conjugates. Polymerization synthesis was capable of exceeding 90% monomer conversion in 30 min. at a reaction temperature of 25 °C, which demonstrates it as a rapid technique [44]. This process of HRP initiated RAFT is shown in Scheme 1, with the enzymatic radical generation shown in the top of the scheme, and the RAFT degenerative transfer used to control the reaction shown in the bottom of the scheme.

The advantages of HRP-catalyzed RAFT polymerization can be optimized through a more thorough understanding of its reaction processes. The specific mechanism and reaction kinetics of HRP-catalyzed polymerization have yet to be fully explored and are the focus of this study. Zhang et al. reported pseudo-first order kinetics, which are observed due the semilogarithmic conversion of polymer being linearly related to time [41]. Previous studies have focused on the kinetics of HRP assays and have demonstrated the inhibitory effects of hydrogen peroxide on HRP activity [45,48]. Our earlier study included a brief report of how HRP activity is affected by the radical transfer components CTA and ACAC when included in the assay. However, the scope of the previous study was limited in that all HRP-catalyzed polymerizations were run under the same conditions and the effect of altering reaction conditions such as reactant concentration was not investigated. This study examines how the reaction rate of HRP-catalyzed RAFT polymerization is affected when the reaction components HRP, CTA, hydrogen peroxide, and ACAC are varied. In this work, a detailed kinetic study is performed in each component of the HRP catalyzed RAFT process. The target of this work is to correlate the observed polymerization reaction kinetics to the underlying enzymatic activity, guiding how to optimize the HRP-catalyzed RAFT polymerization.

## 2. Experimental

### 2.1. Materials

All materials were purchased from commercial suppliers unless otherwise specified. All materials were used as received unless otherwise specified. 2,4-pentadione or acetylacetone (Alfa Aesar, acac, Tewksbury, MA, USA) was used as received. *N*,*N*-dimethylacrylamide (DMAm, Acros Organics, Tewksbury, MA, USA) was passed over a short column of basic alumina to remove the inhibitor. Horseradish peroxidase type I powder (HRP, 146 units/mg, Sigma, Burlington, VT, USA) was stored at 4 °C. (2-propionic acid)yl ethyl trithiocarbonate (PAETC) was synthesized following procedures described in the literature [49,50]. 

### 2.2. Typical HRP Catalyzed RAFT Polymerization of DMAm

A clean 10 mL round bottom flask was used as the reaction vessel. DMAm (72.2 mg, 729 μmol), a stock solution of PAETC in 20 mM acetate buffer at pH = 5.5 (1 mL, 1.53 mg PAETC, 7.29 μmol PAETC), and pH 5.5, 20 mM acetate buffer (3 mL) were added initially. This solution was deoxygenated by bubbling nitrogen gas through it for 10 min. A 2.7% solution of H_2_O_2_ (14 μL, 0.42 mg H_2_O_2_, 12.3 μmol H_2_O_2_) and ACAC (7 μL, 6.9 mg ACAC, 69 μmol ACAC) were added to the reaction solution. A 15 mg/mL stock solution of HRP was then prepared in pH 5.5, 20 mM acetate buffer. 200 μL of this HRP solution was added to the reaction mixture. The reaction mixture was gently stirred at 25 °C and samples of approximately 100 μL were taken periodically to monitor the polymerization progress. Each sample was immediately exposed to oxygen and then frozen in liquid nitrogen to terminate any polymerization progress. The samples were analyzed after thawing. NMR analysis of samples was conducted by transferring 25–40 μL of sample to approximately 0.5 mL of D_2_O. NMR was used to measure monomer conversion using D_2_O as solvent. GPC analysis of samples was conducted by transferring 25–40 μL of sample to 2 mL of DMF + 0.1% LiBr. Typical variations included changing the concentration of HRP, H_2_O_2_, PAETC, and ACAC.

### 2.3. Typical HRP Activity Assay

The activity of HRP was determined using a method adapted from the literature [48]. A 4-aminoantipyrine solution (AAP)/phenol (PhOH) working solution was prepared with 10 mL of 0.1% AAP (1 mg/mL, in water), 20 mL 0.1% PhOH solution (1 mg/mL in water), and 70 mL 20 mM phosphate buffer, pH = 6. A 14.7 mM H_2_O_2_ stock solution was prepared. A stock solution of 20 μg/mL HRP in 20 mM phosphate buffer, pH = 6 was prepared. 900 μL of working solution of AAP and PhOH, 50 μL of 0.0147 M H_2_O_2_ solution, and 50 μL of 20 μg/mL HRP solution were added to a cuvette. The absorbance change was then measured at 500 nm over 30 s and a slope was recorded in absorbance/min. Typical variations included different loadings of HRP, PAETC, H_2_O_2_, and ACAC.

### 2.4. NMR

All nuclear magnetic resonance (NMR) was performed on a Bruker 500 MHz spectrometer (Billerica, MA, USA). 

### 2.5. UV-Visible Spectroscopy

HRP activity assays were measured on a Spectronic Genesys 5 spectrophotometer (Waltham, MA, USA), taking measurements at 500 nm.

### 2.6. Size Exclusion Chromatography (SEC)

Size exclusion chromatography (SEC) was performed on an Agilent SEC system (Waldbronn, Germany) comprised of an Agilent 1260 isocratic pump, an Agilent autosampler, 1 × Agilent PolarGel-M-guard and 2 × Agilent PolarGel-M analytical columns and an Agilent 1260 refractive index (RI) detector. *N*,*N*-dimethylformamide (DMF) with 0.1 wt % LiBr was the eluent at a flow rate of 1 mL/min, maintained at 50 °C. The system was calibrated with poly(methyl methacrylate) (PMMA) standards with molecular weights the range of 617,500 to 1010. Each sample was filtered through a PTFE 200 nm filter.

## 3. Results

The focus of this study is to probe the underlying reaction mechanism in RAFT polymerization using HRP as a RAFT-initiase. A combination of polymerization kinetic analysis as well as enzymatic assays was used to probe the underlying reaction process, and to provide guidelines for optimization and implementation in future studies. 

**Figure 1:**
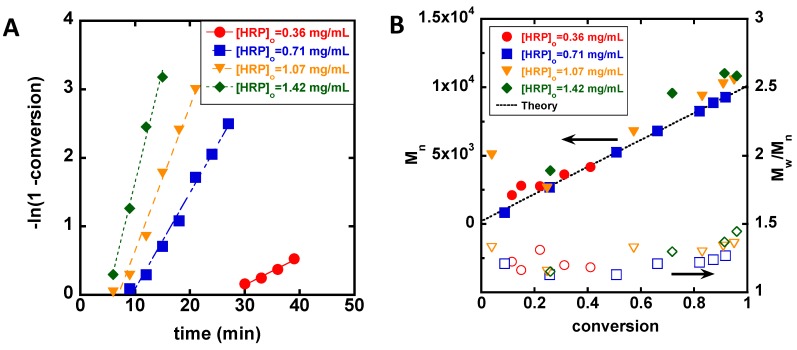
(**A**) Semilogarithmic plots and (**B**) evolution of M_n_ (solid points) and M_w_/M_n_ (open points) of HRP-catalyzed polymerization. Red points (circles) show [HRP]_0_ = 0.36 mg/mL. Blue points (squares) show [HRP]_0_ = 0.71 mg/mL. Orange points (triangles) show [HRP]_0_ = 1.07 mg/mL. Green points (diamonds) show [HRP]_0_ = 1.42 mg/mL. Assays were conducted with component ratios of [DMAm]_0_:[PAETC]_0_:[ACAC]_0_:[H_2_O_2_]_0_ = 100:1:9.7:1.7, and concentrations of [DMAm] = 170 mM, [HRP] = **X** mg/mL in 4.2 mL of pH = 5.5, 20 mM acetate buffer at 25 °C.

Since this process is enzymatically initiated, the impact of the horseradish peroxidase concentration was initially investigated. As expected in an enzymatically-catalyzed process, higher concentration of enzyme led to increased reaction rates. Figure 1A shows linear semi-logarithmic plots at each HRP concentration with the induction decreasing with the higher enzyme loadings. The final conversion at lower HRP concentrations was lower as well. Figure 1B indicates good agreement between theoretical and experimental M_n_ values and acceptable molar mass disparities, (M_w_/M_n_ typically less than 1.40).

**Figure 2:**
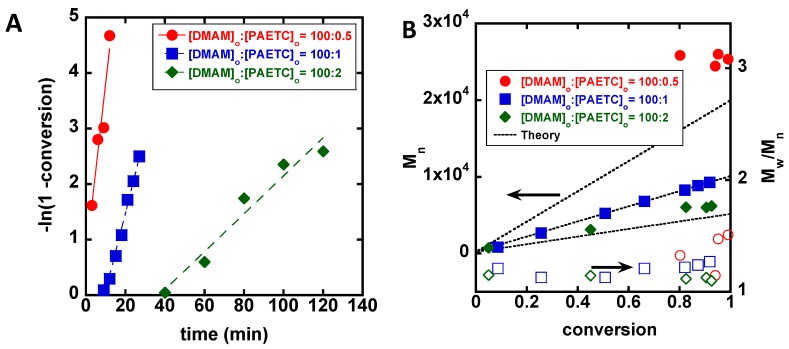
(**A**) Semilogarithmic plots and (**B**) evolution of M_n_ (solid points) and M_w_/M_n_ (open points) of HRP-catalyzed polymerization. Red points (circles) show [DMAM]_0_:[PAETC]_0_ = 100:0.5. Blue points (squares) show [DMAM]_0_:[PAETC]_0_ = 100:1. Green points (diamonds) show [DMAM]_0_:[PAETC]_0_ = 100:2. Assays were conducted with component ratios of [DMAm]_0_:[PAETC]_0_:[ACAC]_0_:[H_2_O_2_]_0_ = 100:**X**:9.7:1.7, and concentrations of [DMAm] = 170 mM, [HRP] = 0.71 mg/mL in 4.2 mL of pH = 5.5, 20 mM acetate buffer at 25 °C.

A key component of enzyme-initiated RAFT is the chain transfer agent (CTA), for which concentration is an important parameter to be investigated. Figure 2A shows the reaction went to full conversion in under ten minutes when [DMAM]_0_:[PAETC]_0_ = 100:0.5, however when [DMAM]_0_:[PAETC]_0_ = 100:2, the reaction was still in progress after 120 min. Lower concentrations of CTA resulted in a less controlled polymerization, as shown in Figure 2B. Agreement between theoretical and experimental M_n_ values was especially poor when [DMAM]_0_:[PAETC]_0_ = 100:0.5. This effect is magnified further when considering the system with even lower CTA loading, i.e., [DMAM]_0_:[PAETC]_0_ = 100:0.25, which is given in Figure S1. This poorer control is correlated with the increased reaction rates, and could be expected if the system with low CTA loading had too high a radical concentration for the amount of the CTA, which is the controlling agent in RAFT systems. It is important to note that in an ideal RAFT polymerization, the concentration of the chain transfer agent should not impact the rate of the reaction, since the process is a degenerative transfer. This suggests that the CTA could be decreasing the polymerization rate through an inhibitory process with the HRP enzyme. This will be further probed when evaluating enzymatic activity.

**Figure 3:**
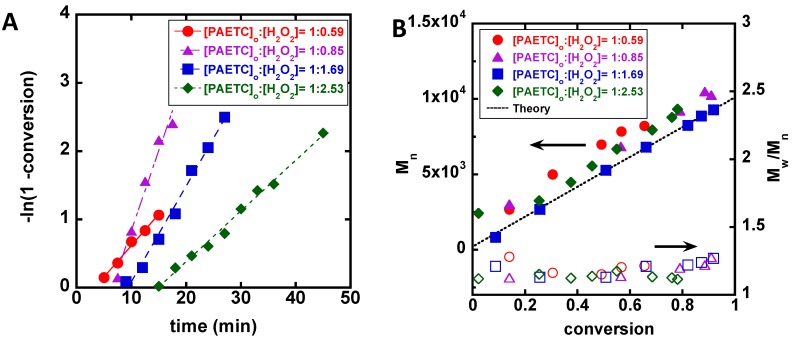
(**A**) Semilogarithmic plots and (**B**) evolution of M_n_ (solid point) and M_w_/M_n_ (open points) of HRP-catalyzed polymerization. Red points (circles) show [PAETC]_0_:[H_2_O_2_] = 1:0.59. Purple points (triangles) show [PAETC]_0_:[H_2_O_2_] = 1:0.85. Blue points (squares) show [PAETC]_0_:[H_2_O_2_] = 1:1.69. Green points (diamonds) show [PAETC]_0_:[H_2_O_2_] = 1:2.53. Component ratios utilized were [DMAm]_0_:[PAETC]_0_:[ACAC]_0_:[H_2_O_2_]_0_ = 100:1:9.7:**X**, and concentrations were [DMAm] = 170 mM, [HRP] = 0.71 mg/mL in 4.2 mL of pH = 5.5, 20 mM acetate buffer at 25 °C.

Hydrogen peroxide was then studied as a reaction parameter. Since hydrogen peroxide is the substrate for HRP, a higher concentration of hydrogen peroxide should result in a higher rate of radical generation, which should lead to a higher reaction rate under typical RAFT polymerization conditions. However, hydrogen peroxide is shown to be inhibitory to HRP at higher concentrations (Figure 3A and Figure 7), which results in reduced polymerization reaction rates. Figure 3A shows linear conversion at each hydrogen peroxide concentration. Conversion was reduced when [PAETC]_0_:[H_2_O_2_] = 1:0.59. Figure 3B indicates acceptable agreement between theoretical and experimental M_n_ values and acceptable molar mass disparities, (M_w_/M_n_ typically less than 1.30). The complex behavior of polymerization rate with hydrogen peroxide concentration suggests a dual role for this reagent, one as a substrate and the other as an inhibitor or denaturant of the enzyme. This will be investigated in greater detail when probing the underlying enzymatic activity.

**Figure 4:**
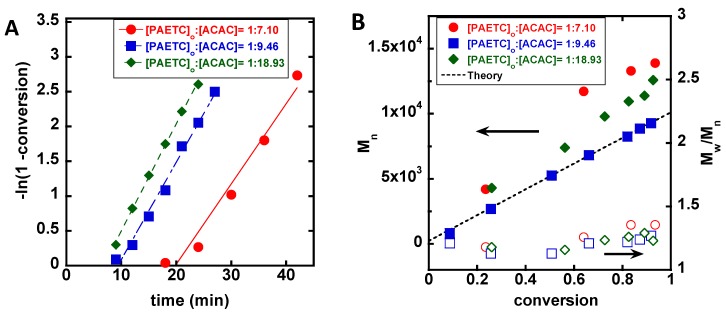
(**A**) Semilogarithmic plots and (**B**) evolution of M_n_ (solid point) and M_w_/M_n_ (open points) of HRP-catalyzed polymerization. Red points (circles) show [PAETC]_0_:[ACAC] = 1:7.10. Blue points (squares) show [PAETC]_0_:[ACAC] = 1:9.46. Green points (diamonds) show [PAETC]_0_:[ACAC] = 1:18.93. Component ratios utilized were [DMAm]_0_:[PAETC]_0_:[ACAC]_0_:[H_2_O_2_]_0_ = 100:1:**X**:1.7, and concentrations were [DMAm] = 170 mM, [HRP] = 0.71 mg/mL in 4.2 mL of pH = 5.5, 20 mM acetate buffer at 25 °C.

ACAC was the final reaction parameter investigated as related to kinetics. ACAC acts primarily as a mediator, transferring the OH and heme bound radicals generated by HRP to simple carbon centered radicals that are capable of initiating polymerization. Figure 4A indicates linear reaction progress, with some deviation when [PAETC]_0_:[ACAC] = 1:7.10. No reaction was observed when [ACAC] = 0 mM, which shows that it is an essential component to HRP-catalyzed polymerization. Further, the reaction rate increased, albeit in a diminishing fashion, at higher ACAC concentrations. The kinetic data suggest that there is a critical concentration of the mediator ACAC needed for efficient polymerization. In addition, control over the polymerization was reduced when ACAC concentrations are lowered, as shown in Figure 4B. There is reduced agreement between theoretical and experimental M_n_ values when [PAETC]_0_:[ACAC] = 1:7.10. 

**Figure 5:**
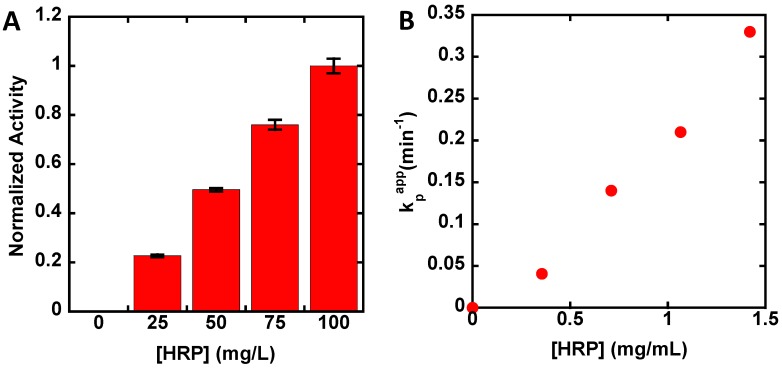
(**A**) Relative rate of enzymatic activity, as a function of HRP loading, measured by the reaction of aminoantipyrine and phenol, normalized to activity at 100 mg/L HRP loading, (**B**) Apparent polymerization rate (k_p_^app^) under the conditions: [DMAm]_0_:[PAETC]_0_:[ACAC]_0_:[H_2_O_2_]_0_ = 100:1:9.7:1.7, [DMAm] = 170 mM, [HRP] = **X** mg/mL in 4.2 mL of pH = 5.5, 20 mM acetate buffer at 25 °C.

In order to investigate whether the observed kinetic trends are due to impacts of the reagent on the HRP enzymatic turn over, or due to down-stream effects on the RAFT process, the polymerization kinetics were correlated with the intrinsic enzymatic assay kinetics as a function of each reaction component. Initially, the impact of enzyme loading was investigated. Figure S2 indicates that the addition of DMAm monomer at the concentrations used in these reactions has negligible impact on the enzymatic activity. As shown in Figure 5, increased enzyme loading led to a higher apparent enzymatic activity, and this correlated well to the apparent rate of polymerization (k_p_^app^). As anticipated, this shows that, as anticipated, higher enzyme concentrations lead to an increase in the rate of radical production, leading to both a faster rate of polymerization, and a higher rate of turnover of phenol/aminoantipyrine to the products. 

**Figure 6:**
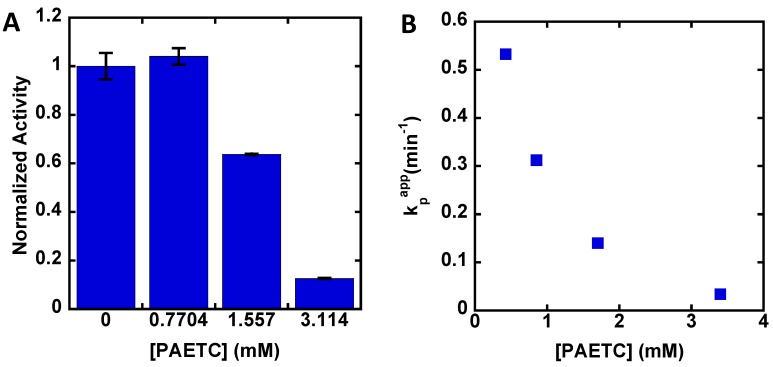
(**A**) Relative rate of enzymatic activity, as a function of PAETC loading, measured by the reaction of aminoantipyrine and phenol, normalized to activity at no PAETC loading; (**B**) Apparent polymerization rate (k_p_^app^) under the conditions: [DMAm]_0_:[PAETC]_0_:[ACAC]_0_:[H_2_O_2_]_0_ = 100:**X**:9.7:1.7, [DMAm] = 170 mM, [HRP] = 0.71 mg/mL in 4.2 mL of pH = 5.5, 20 mM acetate buffer at 25 °C.

Interestingly, when considering the chain transfer agent, PAETC, the polymerization kinetics in Figure 2 indicated a decrease in rate. To determine the origin of this reduction in polymerization rate, HRP enzymatic assays were performed in conjunction with the polymerization results. As indicated in Figure 6, low concentrations of PAETC had minimal impact on the enzymatic activity or polymerization rate, however, higher concentrations of the PAETC RAFT agent led to substantial decreases in the enzymatic activity, both as the ability to react phenol with aminoantipyrine as well as the enzyme’s ability to act as a RAFT initiase. These data suggest that the PAETC is perturbing the enzyme, possibly at the active site, decreasing its ability to act as an initiator for RAFT polymerization. Nevertheless, non-zero polymerization rate and enzymatic activity is observed in all cases studied. This indicates that molecular weight can be effectively controlled by the enzymatic RAFT process, although lower targeted molecular weights will typically lead to slower polymerizations.

**Figure 7:**
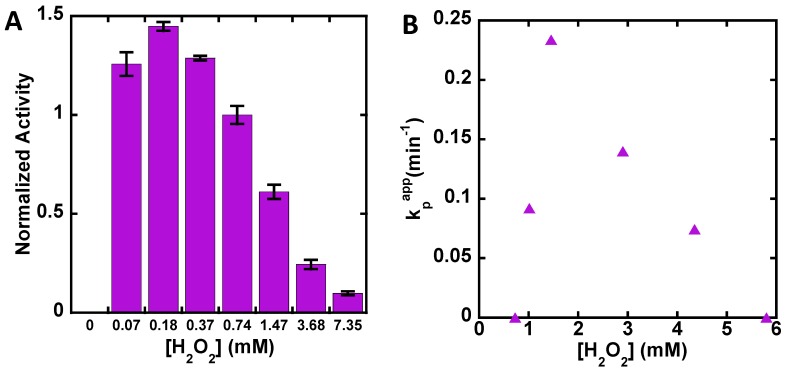
(**A**) Relative rate of enzymatic activity, as a function of H_2_O_2_ loading, measured by the reaction of aminoantipyrine and phenol, normalized to activity at [H_2_O_2_] = 0.74 mM loading; (**B**) Apparent polymerization rate (k_p_^app^) under the conditions: [DMAm]_0_:[PAETC]_0_:[ACAC]_0_:[H_2_O_2_]_0_ = 100:1:9.7:**X**, [DMAm] = 170 mM, [HRP] = 0.71 mg/mL in 4.2 mL of pH = 5.5, 20 mM acetate buffer at 25 °C.

The substrate of HRP is H_2_O_2_, which would intuitively suggest that higher loadings of H_2_O_2_ should lead to increased enzymatic activity and polymerization. However, as displayed in Figure 7, there is an optimal loading of H_2_O_2_ near 0.2–0.4 mM in the enzymatic assays and 1 mM in the polymerization experiments, that gives highest performance of the enzyme. At low H_2_O_2_ loading the turnover rate is low, presumably due to the low substrate concentration, while at high H_2_O_2_ loading the enzyme could be deactivated by the highly reactive H_2_O_2_ substrate [48]. This indicates that there is an optimal concentration of H_2_O_2_ that gives a balance between enzymatic stability and performance with loading of the peroxide substrate.

**Figure 8:**
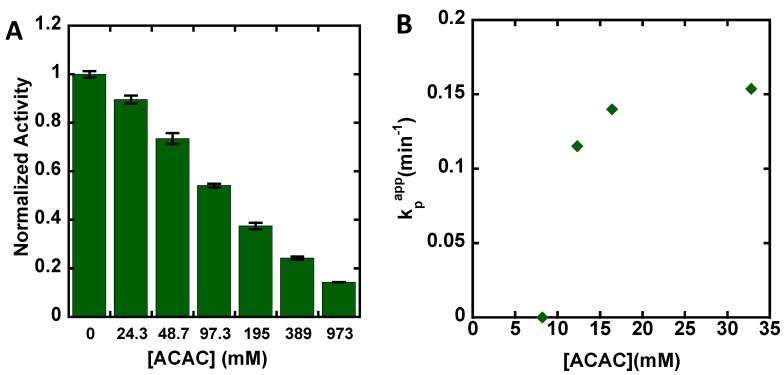
(**A**) Relative rate of enzymatic activity, as a function of ACAC loading, measured by the reaction of aminoantipyrine and phenol, normalized to activity at no ACAC loading; (**B**) Apparent polymerization rate (k_p_^app^) under the conditions: [DMAm]_0_:[PAETC]_0_:[ACAC]_0_:[H_2_O_2_]_0_ = 100:1:**X**:1.7, [DMAm] = 170 mM, [HRP] = 0.71 mg/mL in 4.2 mL of pH = 5.5, 20 mM acetate buffer at 25 °C.

The final parameter to be investigated is the loading of the ACAC mediator. Kinetic analysis in Figure 8 indicates that the k_p_^app^ value increases and eventually plateaus when loadings of ACAC are increased. In contrast the apparent enzymatic activity, as measured by the rate of reaction of phenol with aminoantipyrine, decreases with increasing ACAC loading. The results in Figure 8 are unlike the other assays, where apparent enzymatic activity correlated well with polymerization rate. However, it is important to note that ACAC is a mediator of the polymerization and serves to generate carbon centered radicals from the highly reactive hydroxyl and heme centered radicals generated through the HRP catalytic cycle. Therefore, at a sufficiently high ACAC loading, essentially all radicals generated by HRP will react with ACAC to create carbon centered radicals capable of initiating polymerization, leading to a plateau in rate with ACAC loading. However, by the same mechanism, at higher ACAC loadings, ACAC derived radicals will be generated, rather than the product of phenol with aminoantipyrine, leading to a decrease in apparent enzymatic activity. Therefore, the reduction in apparent HRP activity in Figure 8A provides evidence for ACAC acting as a mediator in enzymatic RAFT polymerization.

## 4. Discussion

The key results of the earlier analysis are that enzymatic activity correlates well with polymerization efficiency and rate. The majority of systems displayed a short induction period, which could be due to residual oxygen or the catalase-like activity of HRP in the presence of the H_2_O_2_ substrate [51]. Note that to minimize freezing induced denaturation of the enzyme [52,53], freeze pump thaw cycles are not performed on the enzyme containing solutions, which could lead to traces of residual oxygen. Nevertheless, rapid polymerization typically occurs after this short induction period. This indicates that polymerization kinetics are primarily dictated by enzymatic performance. However, it is important to discuss each result and explain any counterintuitive observations. If operating under simple Michaelis-Menten kinetics, it would be expected that increasing the concentration of HRP for the HRP-catalyzed polymerization should lead to a square root scaling in apparent propagation rates with enzyme loading. This is because the linear increase in radical generation, would also lead to an increase in radical termination, leading to an overall square root scaling [46,54]. However, this type of trend is not observed in Figure 5B, which suggests that the polymerization system is more complicated than a simple Michaelis-Menten kinetics system coupled with free radical polymerization kinetics. This can be attributed to the inhibitory effect of the CTA [55], since higher loadings of HRP may lead to a much larger fraction of enzymatically active protein, at the same CTA concentration. Indeed, Figure 6A show that there is a threshold ratio of CTA:HRP before any inhibition of HRP by the CTA is observed. Sulfur containing molecules are well documented inhibitors of HRP [55]. It is important to note that in all systems, the enzyme concentration is lower than the CTA concentration showing that the CTA does not quantitatively inhibit the HRP enzyme, suggesting a relatively weak or reversible mode of inhibition. When the concentrations of CTA:HRP are below that threshold ratio, no inhibition is observed. This would suggest that the trend in reaction rate vs. HRP concentration would have two distinct forms. A linear like trend similar to what is seen in Figure 5B would be expected on lower HRP concentration, where the CTA:HRP ratio is higher. Concentrations of HRP greater than this would be when the CTA:HRP ratio is greater than the threshold ratio, which represents fewer HRP being inhibited by the CTA. 

The decreasing trend of apparent HRP activity when concentrations of ACAC are increased (as shown in Figure 8A) can be explained by ACAC competing with 4-aminoantipyrine for HRP-produced radicals, which lowers the amount of oxidized 4-aminoantipyrine produced. The decrease in normalized activity corresponds to the amount of radicals reacting with ACAC [44]. These results show that increased concentrations of ACAC will produce more radicals from hydrogen peroxide, however the effect is lowered at higher concentrations. This can be explained by ACAC being in excess at these higher concentrations and is reacting with the hydrogen peroxide near its maximum rate. Figure 8B shows a complementary result that, as the concentration of ACAC approaches 20 mM, there is a sufficient amount of ACAC to match the rate of HRP-catalyzed hydrogen peroxide radicals, so the overall reaction rate increases marginally despite the much higher increase in ACAC concentration.

Hydrogen peroxide is known to reversibly inhibit HRP at high concentrations [48]. However, it also acts as the substrate and follows basic enzyme kinetics, to a certain extent, where increasing the substrate will lead to an increase in catalysis rate. HRP also has catalase [51], or oxygen evolving, activity against H_2_O_2_, which could decrease the rate of the reaction. Therefore, H_2_O_2_ has a competitive roles in the polymerization acting as both an inhibitor at high concentrations as well as a necessary substrate in order to generate the radicals [56]. Figure 7A,B show hydrogen peroxide having both a positive and negative effect on HRP catalysis. Both the activity assay system and the HRP-catalyzed polymerization system had maximum reaction rates when hydrogen peroxide concentrations were around 0.2–1 mM, with differences likely due to the enzyme and the different ratios of H_2_O_2_ to HRP ([HRP] = 0.020 mg/mL for the activity assays and [HRP] = 0.71 mg/mL for the polymerization reaction). When considering the ratio of HRP to H_2_O_2_ where inhibition occurs, these data suggest that hydrogen peroxide has a greater inhibitory effect when HRP is acting in the polymerization system. This is most likely due to the polymerization reaction requiring a greater amount of radicals for the reaction to proceed, so increased hydrogen peroxide inhibition will decrease the reaction rate more drastically. In addition, it appears that the induction period increases with higher H_2_O_2_ concentration, possibly due to the background catalase activity of HRP [51].

Lowered concentrations of ACAC and CTA result in reduced polymerization control. This can be attributed to the role of these components in the initiation of the polymerization reaction. For example, when CTA concentration is lowered, HRP is less inhibited which results in a greater amount of radical initiator being produced. Increased initiator concentration is shown to increase the amount of terminated polymeric material, which broads the molecular weight distribution [57]. This explains the poor M_n_ control when [DMAM]_0_:[PAETC]_0_ = 100:0.5 in Figure 2B and Figure S1. Lowered concentrations of ACAC result in less radicals being introduced as a mediator to the polymerization reaction. This leads to insufficient initiation, which is known to result in poor polymerization control [58]. This effect is seen in Figure 4B which shows poor agreement between theoretical and experimental M_n_ values when [PAETC]_0_:[ACAC] = 1:7.10. The effects of insufficient initiation are seen in Figure 4A and Figure 8B as seen by the lower induction time and slightly lower apparent propagation rate. Additionally, at lower PAETC concentrations for the same H_2_O_2_ concentration there could be a non-trivial extent of RAFT agent degradation from the background reactions involving H_2_O_2_ [59]. However, it is important to note that typically elevated temperatures of 70 °C are typically used to remove RAFT end groups using H_2_O_2_ [59], suggesting that at the polymerization conditions minimal loss of RAFT agent should occur [36].

**Scheme 2:**
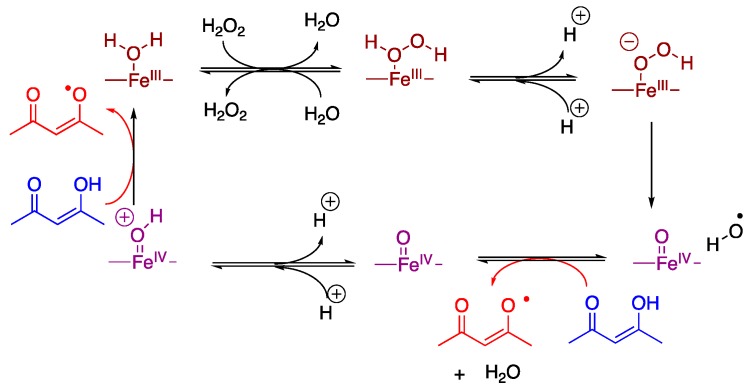
Proposed mechanism for enzymatic cycle in HRP-initiated RAFT polymerization.

Considering the known catalytic cycle of HRP in the presence of H_2_O_2_ [45], a proposed cycle for initiation is developed. This is highlighted in Scheme 2. The key features of this process are that initially, radicals are generated from HRP using H_2_O_2_ as a substrate. However, in the presence of ACAC, a hydrogen atom can be transferred to generate a carbon centered radical that is capable of initiating polymerization in the presence of monomer. In all cases, the role of the RAFT CTA in this mechanism is to facilitate molecular weight control through degenerative transfer, and indeed, higher CTA loadings inhibit the polymerization rate. The kinetic analysis and enzymatic assays in this work are consistent with the role of HRP as a RAFT-initiase, with molecular weight control enabled by the RAFT equilibrium.

## 5. Conclusions

In summary, a detailed investigation into the kinetics of HRP initiated RAFT polymerization was performed. In general, rapid and well-controlled RAFT polymerization could be performed under mild conditions near room temperature. Polymerization rate was greatly enhanced with enzyme loading. The enzyme substrate H_2_O_2_ has a complex impact on polymerization rate, with an optimal concentration near 1 mM under the studied conditions. A critical concentration of the mediator, ACAC, is needed to facilitate initiation of the polymerization, although higher concentrations lead to negligible improvements in the rate of reaction. In order to have controlled polymerization a RAFT CTA is needed, although the RAFT agent used has an inhibitory effect on polymerization rate, however, control over molecular weight is improved at higher CTA loadings. The observed trends in polymerization kinetics are rationalized through careful analysis and comparison to enzymatic activity assays for HRP.

## Supplementary Materials

The following are available online at http://www.mdpi.com/2073-4360/10/7/741/s1, Polymerization kinetics with low chain transfer agent concentrations. Enzymatic assay of HRP in the presence and absence of DMAm monomer.

## References

[1] Schmid A., Dordick J.S., Hauer B., Kiener A., Wubbolts M., Witholt B. (2001). Industrial biocatalysis today and tomorrow. Nature.

[2] Sheldon R.A. (2008). E factors, green chemistry and catalysis: An odyssey. Chem. Commun..

[3] Liu W., Kumar J., Tripathy S., Samuelson L.A. (2002). Enzymatic Synthesis of Conducting Polyaniline in Micelle Solutions. Langmuir.

[4] Uyama H., Kobayashi S. (2002). Enzyme-catalyzed polymerization to functional polymers. J. Mol. Catal. Enzym..

[5] Gross R.A., Kumar A., Kalra B. (2001). Polymer Synthesis by In Vitro Enzyme Catalysis. Chem. Rev..

[6] Kobayashi S., Uyama H., Kimura S. (2001). Enzymatic Polymerization. Chem. Rev..

[7] Kobayashi S., Makino A. (2009). Enzymatic Polymer Synthesis: An Opportunity for Green Polymer Chemistry. Chem. Rev..

[8] Sen S., Puskas E.J. (2015). Green Polymer Chemistry: Enzyme Catalysis for Polymer Functionalization. Molecules.

[9] Moad G., Solomon D.H. (1990). Invited Review. Understanding and Controlling Radical Polymerization. Aust. J. Chem..

[10] Moad G., Solomon D.H. (2006). The Chemistry of Radical Polymerization.

[11] Kalra B., Gross R.A. (2000). Horseradish Peroxidase Mediated Free Radical Polymerization of Methyl Methacrylate. Biomacromolecules.

[12] Sanchez-Leija R.J., Torres-Lubian J.R., Resendiz-Rubio A., Luna-Barcenas G., Mota-Morales J.D. (2016). Enzyme-mediated free radical polymerization of acrylamide in deep eutectic solvents. RSC Adv..

[13] Cai Z.-Q.I., Wang W., Ruan G., Wen X. (2012). Kinetic study of acrylamide radical polymerization initiated by the horseradish peroxidase–mediated system. Int. J. Chem. Kinet..

[14] Durand A., Lalot T., Brigodiot M., Maréchal E. (2000). Enzyme-mediated initiation of acrylamide polymerization: Reaction mechanism. Polymer.

[15] Durand A., Lalot T., Brigodiot M., Maréchal E. (2001). Enzyme-mediated radical initiation of acrylamide polymerization: Main characteristics of molecular weight control. Polymer.

[16] Emery O., Lalot T., Brigodiot M., Maréchal E. (1997). Free-radical polymerization of acrylamide by horseradish peroxidase-mediated initiation. J. Polym. Sci. Part A Polym. Chem..

[17] Hollmann F., Arends I.W.C.E. (2012). Enzyme Initiated Radical Polymerizations. Polymers.

[18] Lalot T., Brigodiot M., Maréchal E. (1999). A kinetic approach to acrylamide radical polymerization by horse radish peroxidase-mediated initiation. Polym. Int..

[19] Shogren R.L., Willett J.L., Biswas A. (2009). HRP-mediated synthesis of starch–polyacrylamide graft copolymers. Carbohydr. Polym..

[20] Singh A., Ma D., Kaplan D.L. (2000). Enzyme-Mediated Free Radical Polymerization of Styrene. Biomacromolecules.

[21] Teixeira D., Lalot T., Brigodiot M., Maréchal E. (1999). β-Diketones as Key Compounds in Free-Radical Polymerization by Enzyme-Mediated Initiation. Macromolecules.

[22] Gormley A.J., Chapman R., Stevens M.M. (2014). Polymerization Amplified Detection for Nanoparticle-Based Biosensing. Nano Lett..

[23] Zavada S., Battsengel T., Scott T. (2016). Radical-Mediated Enzymatic Polymerizations. Int. J. Mol. Sci..

[24] Russell G.T. (2002). The Kinetics of Free-Radical Polymerization: Fundamental Aspects. Aust. J. Chem..

[25] Braunecker W.A., Matyjaszewski K. (2007). Controlled/living radical polymerization: Features, developments, and perspectives. Prog. Polym. Sci..

[26] Goto A., Fukuda T. (2004). Kinetics of living radical polymerization. Prog. Polym. Sci..

[27] Chiefari J., Chong Y.K., Ercole F., Krstina J., Jeffery J., Le T.P.T., Mayadunne R.T.A., Meijs G.F., Moad C.L., Moad G. (1998). Living Free-Radical Polymerization by Reversible Addition-Fragmentation Chain Transfer: The RAFT Process. Macromolecules.

[28] Georges M.K., Veregin R.P.N., Kazmaier P.M., Hamer G.K. (1993). Narrow molecular weight resins by a free-radical polymerization process. Macromolecules.

[29] Kato M., Kamigaito M., Sawamoto M., Higashimura T. (1995). Polymerization of Methyl Methacrylate with the Carbon Tetrachloride/Dichlorotris-(triphenylphosphine)ruthenium(II)/Methylaluminum Bis(2,6-di-tert-butylphenoxide) Initiating System: Possibility of Living Radical Polymerization. Macromolecules.

[30] Wang J.-S., Matyjaszewski K. (1995). Controlled/“living” radical polymerization. atom transfer radical polymerization in the presence of transition-metal complexes. J. Am. Chem. Soc..

[31] Ng Y.-H., di Lena F., Chai C.L.L. (2011). Metalloenzymatic radical polymerization using alkyl halides as initiators. Polym. Chem..

[32] Ng Y.-H., di Lena F., Chai C.L.L. (2011). PolyPEGA with predetermined molecular weights from enzyme-mediated radical polymerization in water. Chem. Commun..

[33] Silva T.B., Spulber M., Kocik M.K., Seidi F., Charan H., Rother M., Sigg S.J., Renggli K., Kali G., Bruns N. (2013). Hemoglobin and Red Blood Cells Catalyze Atom Transfer Radical Polymerization. Biomacromolecules.

[34] Simakova A., Mackenzie M., Averick S.E., Park S., Matyjaszewski K. (2013). Bioinspired Iron-Based Catalyst for Atom Transfer Radical Polymerization. Angew. Chem. Int. Ed..

[35] Sigg S.J., Seidi F., Renggli K., Silva T.B., Kali G., Bruns N. (2011). Horseradish Peroxidase as a Catalyst for Atom Transfer Radical Polymerization. Macromol. Rapid Commun..

[36] Chapman R., Gormley A.J., Herpoldt K.-L., Stevens M.M. (2014). Highly Controlled Open Vessel RAFT Polymerizations by Enzyme Degassing. Macromolecules.

[37] Enciso A.E., Fu L., Russell A.J., Matyjaszewski K. (2018). A Breathing Atom-Transfer Radical Polymerization: Fully Oxygen-Tolerant Polymerization Inspired by Aerobic Respiration of Cells. Angew. Chem. Int. Ed..

[38] Yeow J., Chapman R., Gormley A.J., Boyer C. (2018). Up in the air: Oxygen tolerance in controlled/living radical polymerisation. Chem. Soc. Rev..

[39] Liu Z., Lv Y., An Z. (2017). Enzymatic Cascade Catalysis for the Synthesis of Multiblock and Ultrahigh-Molecular-Weight Polymers with Oxygen Tolerance. Angew. Chem..

[40] Hill M.R., Carmean R.N., Sumerlin B.S. (2015). Expanding the Scope of RAFT Polymerization: Recent Advances and New Horizons. Macromolecules.

[41] Zhang B., Wang X., Zhu A., Ma K., Lv Y., Wang X., An Z. (2015). Enzyme-Initiated Reversible Addition–Fragmentation Chain Transfer Polymerization. Macromolecules.

[42] Liu Z., Lv Y., Zhu A., An Z. (2018). One-Enzyme Triple Catalysis: Employing the Promiscuity of Horseradish Peroxidase for Synthesis and Functionalization of Well-Defined Polymers. ACS Macro Lett..

[43] Tan J., Xu Q., Li X., He J., Zhang Y., Dai X., Yu L., Zeng R., Zhang L. (2018). Enzyme-PISA: An Efficient Method for Preparing Well-Defined Polymer Nano-Objects under Mild Conditions. Macromol. Rapid Commun..

[44] Danielson A.P., Van-Kuren D.B., Lucius M.E., Makaroff K., Williams C., Page R.C., Berberich J.A., Konkolewicz D. (2016). Well-Defined Macromolecules Using Horseradish Peroxidase as a RAFT Initiase. Macromol. Rapid Commun..

[45] Rodríguez-López J.N., Lowe D.J., Hernández-Ruiz J., Hiner A.N.P., García-Cánovas F., Thorneley R.N.F. (2001). Mechanism of Reaction of Hydrogen Peroxide with Horseradish Peroxidase:  Identification of Intermediates in the Catalytic Cycle. J. Am. Chem. Soc..

[46] Goto A., Sato K., Tsujii Y., Fukuda T., Moad G., Rizzardo E., Thang S.H. (2001). Mechanism and Kinetics of RAFT-Based Living Radical Polymerizations of Styrene and Methyl Methacrylate. Macromolecules.

[47] Konkolewicz D., Siauw M., Gray-Weale A., Hawkett B.S., Perrier S. (2009). Obtaining Kinetic Information from the Chain-Length Distribution of Polymers Produced by RAFT. J. Phys. Chem. B.

[48] Nicell J.A., Wright H. (1997). A model of peroxidase activity with inhibition by hydrogen peroxide. Enzym. Microb. Technol..

[49] Falatach R., McGlone C., Al-Abdul-Wahid M.S., Averick S., Page R.C., Berberich J.A., Konkolewicz D. (2015). The best of both worlds: Active enzymes by grafting-to followed by grafting-from a protein. Chem. Commun..

[50] Paeth M., Stapleton J., Dougherty M.L., Fischesser H., Shepherd J., McCauley M., Falatach R., Page R.C., Berberich J.A., Konkolewicz D., Kumar C.V. (2017). Chapter Nine—Approaches for Conjugating Tailor-Made Polymers to Proteins. Methods in Enzymology.

[51] Hiner A.N.P., Hernández-Ruiz J., Williams G.A., Arnao M.B., García-Cánovas F., Acosta M. (2001). Catalase-like Oxygen Production by Horseradish Peroxidase Must Predominantly Be an Enzyme-Catalyzed Reaction. Arch. Biochem. Biophys..

[52] Chang B.S., Kendrick B.S., Carpenter J.F. (1996). Surface-Induced Denaturation of Proteins during Freezing and its Inhibition by Surfactants. J. Pharm. Sci..

[53] Franks F. (1985). Biophysics and Biochemistry at Low Temperatures.

[54] Kurek P.N., Kloster A.J., Weaver K.A., Manahan R., Allegrezza M.L., De Alwis Watuthanthrige N., Boyer C., Reeves J.A., Konkolewicz D. (2018). How Do Reaction and Reactor Conditions Affect Photoinduced Electron/Energy Transfer Reversible Addition–Fragmentation Transfer Polymerization?. Ind. Eng. Chem. Res..

[55] Sariri R., Sajedi R.H., Jafarian V. (2006). Inhibition of horseradish peroxidase activity by thiol type inhibitors. J. Mol. Liquids.

[56] Konkolewicz D., Krys P., Matyjaszewski K. (2014). Explaining Unexpected Data via Competitive Equilibria and Processes in Radical Reactions with Reversible Deactivation. Acc. Chem. Res..

[57] Vana P., Davis T.P., Barner-Kowollik C. (2002). Kinetic Analysis of Reversible Addition Fragmentation Chain Transfer (RAFT) Polymerizations: Conditions for Inhibition, Retardation, and Optimum Living Polymerization. Macromol. Theory Simul..

[58] Mueller A.H., Zhuang R., Yan D., Litvinenko G. (1995). Kinetic analysis of “living” polymerization processes exhibiting slow equilibria. 1. Degenerative transfer (direct activity exchange between active and “dormant” species). Application to group transfer polymerization. Macromolecules.

[59] Jesson C.P., Pearce C.M., Simon H., Werner A., Cunningham V.J., Lovett J.R., Smallridge M.J., Warren N.J., Armes S.P. (2017). H_2_O_2_ Enables Convenient Removal of RAFT End-Groups from Block Copolymer Nano-Objects Prepared via Polymerization-Induced Self-Assembly in Water. Macromolecules.

