# Supplementary material for: Investigating the Mechanism of Horseradish Peroxidase as a RAFT-Initiase
Source: Polymers (Basel). 2018 Jul 5;10(7):741. doi: 10.3390/polym10070741 (PMC6403633; doi:10.3390/polym10070741)
Supplement: Supplementary file 1 [file polymers-10-00741-s001.pdf]

Supporting Information: Investigating the Mechanism of horseradish-peroxide as a RAFT-initiase

Alex P Danielson, Dylan Bailey-Van Kuren, Joshua P. Bornstein, Caleb T. Kozuszek, Jason A. Berberich, Richard C. Page, Dominik Konkolewicz

Supplemental Figures

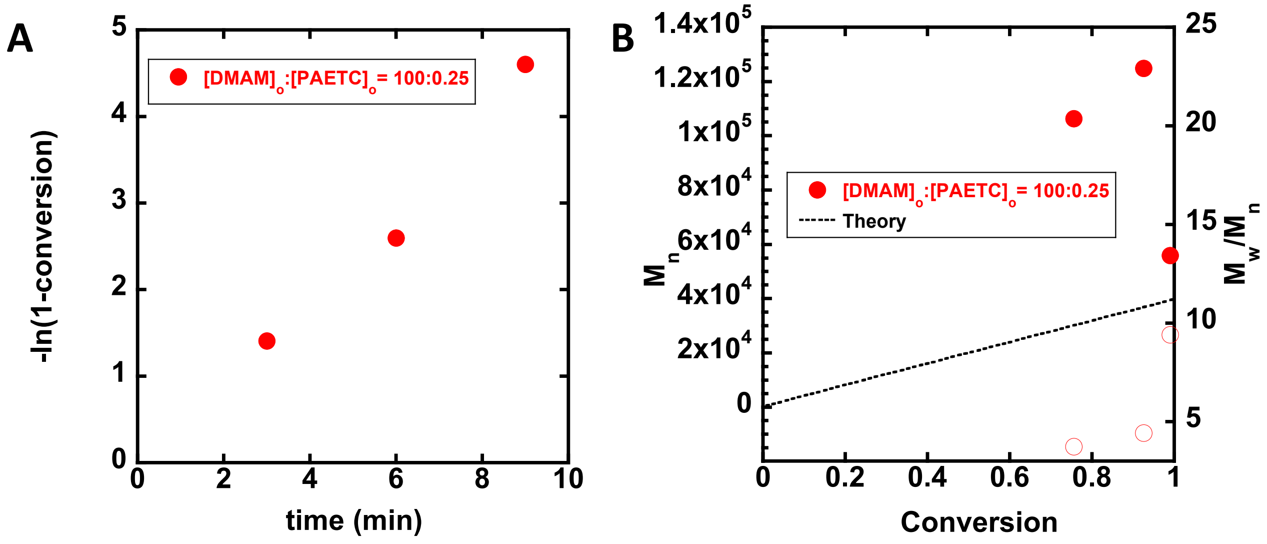

Figure S1: (A) Semilogarithmic plots and (B) evolution of M_n (solid points) and M_w/M_n (open points) of HRP-catalyzed polymerization: $[DMAm]_0$:$[PAETC]_0$:$[ACAC]_0$:$[H_2O_2]_0$= 100:0.25:9.7:1.7, [DMAm]=170 mM, [HRP]= 0.71 mg/mL in 4.2 mL of pH=5.5, 20 mM acetate buffer at 25 °C.

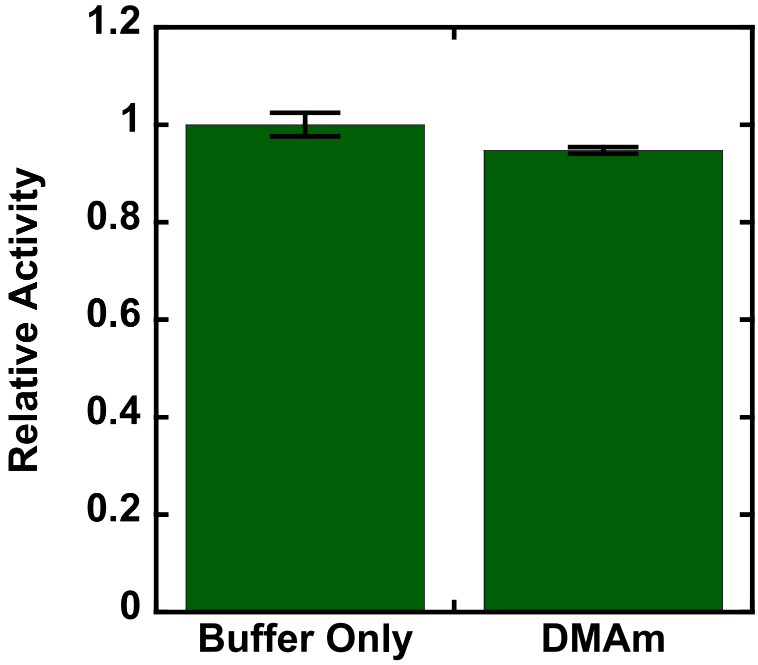

Figure S2: HRP Enzymatic assay and relative activity in the absence and presence of 170 mM DMAm.
